# Magnitude of self-reported intimate partner violence against pregnant women in Ghana’s northern region and its association with low birth weight

**DOI:** 10.1186/s12884-023-06229-6

**Published:** 2024-01-04

**Authors:** Mary Rachael Kpordoxah, Abraham Awonboro Adiak, Abdul-Nasir Issah, Daudi Yeboah, Nashiru Abdulai, Michael Boah

**Affiliations:** 1 Department of Global and International Health, School of Public Health, University for Development Studies, Tamale, Ghana; 2 Tamale Teaching Hospital, Tamale, P.O. Box TL 16, Ghana; 3 Department of Health Services, Policy, Planning, Management, and Economics, School of Public Health, University for Development Studies, Tamale, Ghana; 4 Department of Epidemiology, Biostatistics, and Disease Control, School of Public Health, University for Development Studies, Tamale, Ghana; 5 Nanton District Assembly, Tamale, P.O. Box 1, Ghana

**Keywords:** Violence, Pregnant women, Pregnancy outcomes, Northern region, Ghana

## Abstract

**Background:**

Low birth weight (LBW) rates are high in the northern region of Ghana, as is tolerance for intimate partner violence (IPV). However, the relationship between the two incidents has not been established. This study assessed the magnitude of IPV against pregnant women and its association with LBW in the northern region of Ghana.

**Methods:**

A cross-sectional study was conducted among 402 postnatal women from five public health care facilities in the Tamale Metropolitan Area, northern Ghana. Data were collected electronically during face-to-face interviews. Validated methods were used to determine IPV exposure during pregnancy and birth weight. Multivariable logistic regression was used to identify the independent association between prenatal exposure to IPV and LBW.

**Results:**

Of the 402 women, 46.5% (95% CI: 41.7, 51.4) experienced IPV during their most recent pregnancy. Of these, 34.8% were psychologically abused, 24.4% were sexually abused, and 6.7% were physically abused. Prenatal IPV exposure was found to be significantly associated with birth weight. Low birth weight was twice as likely among exposed women as among unexposed women (AOR = 2.42; 95% CI: 1.12, 5.26, *p* < 0.05). Low birth weight risk was also higher among women with anaemia in the first trimester (AOR = 3.47; 95% CI: 1.47, 8.23, *p* < 0.01), but was lower among women who made at least four antenatal care visits before delivery (AOR = 0.35; 95% CI: 0.14, 0.89, *p* < 0.05) and male newborns (AOR = 0.23; 95% CI: 0.11, 0.49, *p* < 0.001).

**Conclusion and recommendation:**

IPV during pregnancy is prevalent in the research population, with psychological IPV being more widespread than other kinds. Women who suffered IPV during pregnancy were more likely to have LBW than those who did not. It is essential to incorporate questions about domestic violence into antenatal care protocols. In particular, every pregnant woman should be screened for IPV at least once during each trimester, and those who have experienced violence should be closely monitored for weight gain and foetal growth in the study setting to avert the LBW associated with IPV.

**Plain English summary:**

In the northern region of Ghana, the number of babies born with low birth weight is high, as is the number of adults who are willing to put up with intimate partner violence. However, there has not been any proof that these two incidents are connected. This study looked at how frequently intimate partner violence occurs among pregnant women and how it is linked to low birth weight in northern Ghana’s Tamale Metropolitan Area. A cross-sectional study was conducted with 402 postnatal women from five public health care facilities in the study setting. Information on exposure to intimate partner violence during pregnancy and the birth weight of babies was collected electronically during face-to-face interviews. The study found that of the 402 women, 46.5% had experienced violence by an intimate partner during their most recent pregnancy. Out of these, 34.8% were abused psychologically, 24.4% were abused sexually, and 6.7% were abused physically. Women who were abused were more likely than those who were not to have babies with low birth weight. We concluded that intimate partner violence is common during pregnancy in the study setting and that more women suffered psychological intimate partner violence than other types of violence. Intimate partner violence during pregnancy was linked to low birth weight in the study setting. It is important for antenatal care plans to include questions about intimate partner violence. In particular, every pregnant woman should be assessed for intimate partner violence at least once during each trimester for monitoring.

**Supplementary Information:**

The online version contains supplementary material available at 10.1186/s12884-023-06229-6.

## Background

The international community at the 1993 World Conference on Human Rights and Declaration on the Elimination of Violence Against Women acknowledged that violence against women is a vital public health, social policy, and human rights concern, given the high prevalence of IPV in all regions of the world and its associated short- and long-term health impacts on women’s exposures to it [1]. Every woman is entitled to live a safe and free life devoid of violence, but it is estimated that more than 1 in 4 (27%) ever-partnered women aged 15 or older have experienced intimate partner violence (IPV) at some point in their lives in 2018 [2]. Regrettably, women have acceptable attitudes towards domestic violence against themselves. A study in Africa reported that close to 30% of women endorse wife-beating under certain circumstances, with women in Malawi (17%) having the lowest tolerable attitudes towards domestic violence compared to 30% in Ghana and the highest of 82.3% in Mali [3].

Varying rates of IPV during pregnancy have been reported. A study that assessed the socio-ecological factors of IPV during pregnancy in Ethiopia showed that 44% of women were exposed to IPV during pregnancy [4]. A related study in the same country reported a prevalence of 21% [5]. In another study conducted in South Africa, the authors found 21.35% of women had experienced IPV during pregnancy [6]. A study of six European countries reported a prevalence of any abuse of 34.8% among pregnant women [7]. Differing methodologies, IPV measures, and context may partially explain these differences in rates. Nonetheless, IPV during pregnancy is of particular concern due to the effects it has on the mother and unborn baby. Indeed, maternal complications associated with violence during pregnancy have been acknowledged, including miscarriage, premature rupture of membranes, intrauterine growth restriction (IUGR), placental abruption, and genitourinary tract infections [8–11]. Abused pregnant women are also more likely than their counterparts to have poorer psychosocial health and are less likely to use prenatal care services or underutilize these services if they do [12–15].

In relation to newborn outcomes, particularly low birth weight (LBW), there are reports for and against the association between these two incidents. Some scholars have associated IPV during pregnancy with an increased risk of LBW [16–18]. On the other hand, no association has been found by other studies [19–21]. Children born with LBW have a higher risk of adverse health outcomes, such as mortality within 28 days of birth, morbidity, and physical, neurological, and mental impairments [18, 22, 23].

The northern region of Ghana, according to reports, is noted for its prevalence of both forced and child marriages, which serve as significant drivers of IPV [24]. A recent study has also further established that men in this region exhibited higher tolerance for domestic abuse and were more likely to justify violence against women. Specifically, men in the northern region of Ghana were more likely than those from other regions to see wife-beating as a societal norm [25]. In addition to violence, the northern region has the highest prevalence of low birthweight in Ghana, with 30% of babies being born with LBW in this region [26]. In contrast, the prevalence of LBW in other regions of the country ranges from 8 to 23% [27–31]. The high prevalence of LBW in the region has been associated with maternal, child-related, and contextual factors, including poor maternal dietary patterns and practices during pregnancy, high parity, female newborns, twin deliveries, and rural residence [26, 32, 33]. There is a lack of literature on the association between IPV during pregnancy and LBW. As a result, our understanding of the relationship between the high prevalence of LBW in this part of the country and the role of IPV during pregnancy is limited.

The aim of this study is to determine the magnitude of intimate partner violence that reproductive women in Ghana’s northern region experience during pregnancy and its association with unfavourable newborn outcomes, such as LBW. We hypothesise that women who are exposed to IPV during pregnancy are more likely to have LBW newborns in the study’s setting.

## Methods

### Theoretical framework

**Fig. 1:**
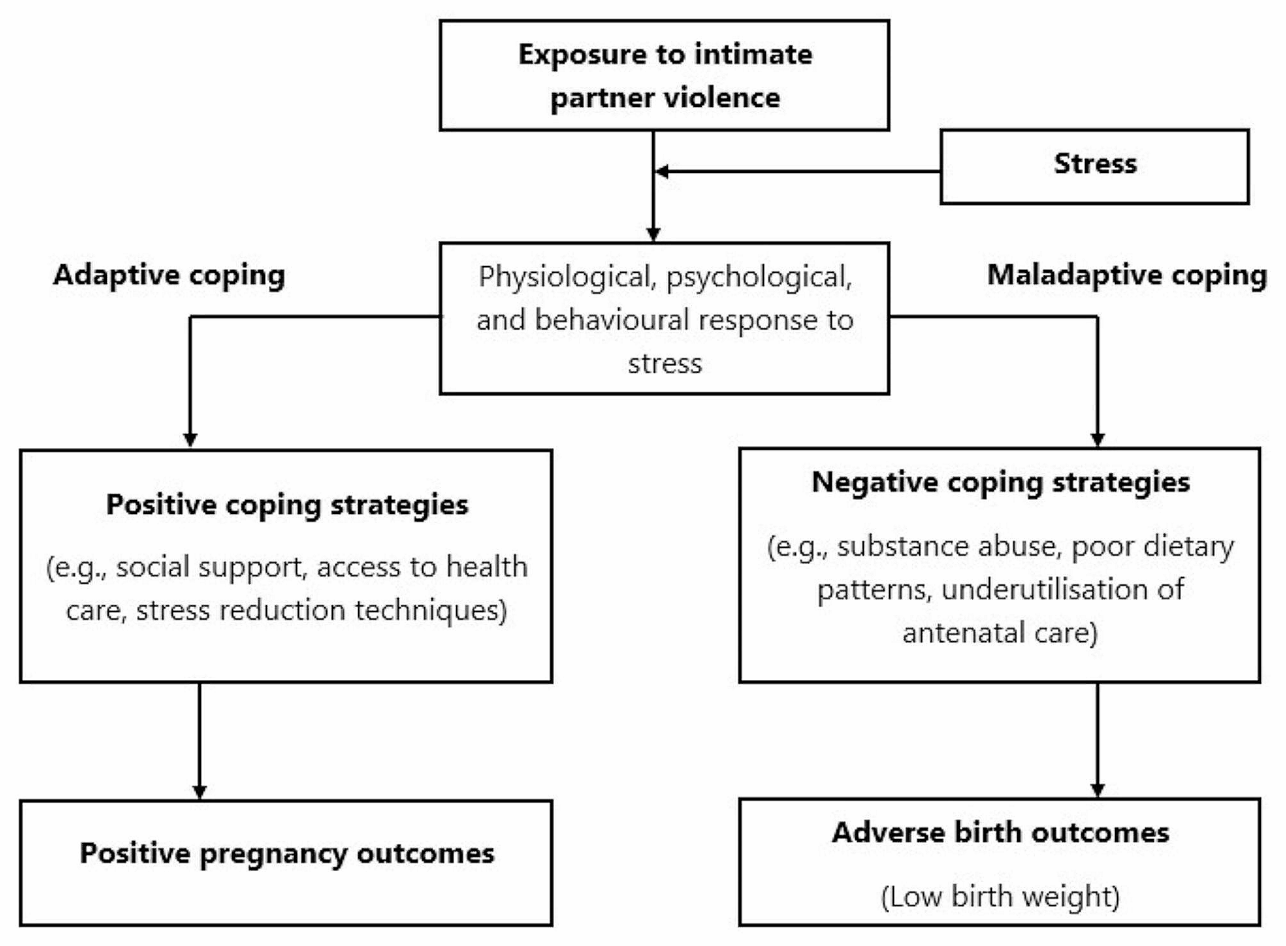
A framework for a plausible explanation linking exposure to intimate partner violence with low birth weight among women of reproductive age. (Adapted from [36])

The exact processes by which prenatal IPV influences birth weight may not be clearly understood. However, we can provide several reasonable explanations for the two incidents. Poor newborn outcomes may occur from prenatal complications caused by direct assault. Pregnancy-related complications such as premature rupture of membranes and placental injury are common among women who have been physically abused during pregnancy and have been linked to poor newborn outcomes such as LBW [11, 34, 35]. We can also explain the link between exposure to IPV during pregnancy and LBW using the “Stress and Coping Model” (Fig. 1). This model is rooted in the field of psychology and focuses on how stressors, including exposure to IPV, and the physiological, behavioural, and psychological responses to the stressors can affect maternal and foetal well-being during pregnancy [36]. In particular, adverse newborn outcomes can potentially result from stress during pregnancy. Stress during pregnancy can cause long-term vasoconstriction of placental blood vessels due to increased cytokines and norepinephrine levels, slowing foetal growth in utero [37]. Stress can also have an impact on a pregnant woman’s dietary habits. Poor nutrition during pregnancy can increase the risk of anaemia, insufficient weight, and IUGR, as well as exacerbate underlying medical conditions and disorders such as eclampsia, which increases the risk of delivering LBW babies [38–42]. IPV exposure during pregnancy can potentially influence a woman’s prenatal care behaviours. Exposed women are substantially more likely to avoid or underutilize antenatal care (ANC), limiting their ability to get preventative therapies such as iron and folic acid supplementation to promote better neonatal health before delivery [43–46]. In sum, our framework identified the physiological and behavioural pathways through which prenatal exposure to IPV can lead to LBW among reproductive women in the study’s setting.

### Study setting and design

This is a facility-based cross-sectional study of women who delivered singletons in five health facilities (Datoyili health centre, Bilpiela health centre, Vittin Reproductive and Child Health, Tamale Central, and Tamale West hospitals) in the Tamale Metropolitan Area, one of sixteen districts in Ghana’s northern region. The research setting serves an estimated population of 374,744 with eighteen community-based health planning and services, eight health centres, and three hospitals, one of which is a teaching hospital.

### Sample size determination and sampling procedures

We used the Taro Yamane formula—$n=\frac{N}{(1+N{e}^{2}) }$–to determine the sample size required for the current study. There were 4,292 expected deliveries from the five facilities selected. Using the expected deliveries (i.e., N), a 5% margin of error (e), and a 10% non-response rate, we estimated that 402 women (n) were required to meet the objective of the study. In the present study, only women who reported being in a union or a partnered relationship and who had given birth, with their child not exceeding 12 months of age, were considered for participation. Multiple sampling procedures were used to select the health facilities and study respondents. Two hospitals were purposefully selected in addition to three health centres that were selected randomly from a pool of eight using the lottery method. The two hospitals are secondary-level facilities that provide ANC, delivery, and postnatal care services to a higher percentage of women in the study setting. The sample required for the study was proportionally distributed to the selected health facilities. Then a systematic random sampling method was used to select respondents from the various facilities. The postnatal registers at the facilities were used for the sampling frame. The respondents were contacted during their attendance at postnatal care services. The interviews continued until the desired sample size for each respective facility was achieved. Data collection tools and procedures

The data were collected electronically through a questionnaire in a face-to-face interview in June 2022. The questionnaire was programmed and administered using the Kobo Collect application on an Android smart phone. The questionnaire was pretested on 10% of the total sample before being used for actual data collection. The forty respondents used for this pretest were recruited from a health centre outside the study’s setting and shared characteristics that were similar to the original study sample. Questions that were found to be ambiguous during the pretest were modified to enhance clarity. The questionnaire gathered information regarding exposure to IPV, age, education, religion, ethnicity, employment, gravidity, gestation at first antenatal care registration and number of visits made before delivery, a malaria episode during pregnancy, haemoglobin levels in the first and second trimesters, birthweight, and sex of the child.

### Variables

#### Dependent variable

The dependent variable considered in the current study was birth weight. We developed two distinct groups for this variable: normal birth weight and low birth weight. The outcome of interest was low birth weight, which was defined as birth weight less than 2500 g (or 2.5 kg). The classifications were based on international standards for the classification of diseases and related health problems [47].

### Explanatory variables

#### Main exposure variable

Prenatal exposure to IPV, the primary exposure variable in the current investigation, was assessed using a 12-item questionnaire adapted from the domestic violence module used in Demographic and Health Surveys (DHS) [48]. Our conception of IPV included physical, psychological, and sexual IPV. There were six yes-or-no questions on acts of physical abuse, three questions on psychological abuse, and three questions on sexual abuse (see Additional file 1) by her current partner during her most recent pregnancy. Exposure was ascertained if a woman answered “yes” to any of the acts of violence, regardless of their frequency or severity. We developed binary measures of physical, psychological, and sexual IPV if a woman reported experiencing one or more of the acts under these categories during her most recent pregnancy. We then created a binary exposure variable, “prenatal exposure to IPV,” to measure whether the woman experienced at least one type of violence during pregnancy. The experience of an act was coded ‘1’ and ‘0’ if otherwise.

### Covariates

In Ghana, several demographic, socioeconomic, and health-related factors can influence birth weight [30, 31, 49]. As a result, we included the following variables as potential confounding factors: sex of child, age of mother, religion, education, employment status, gravida, time of first ANC initiation, number of ANC visits prior to delivery, anaemia in the first and second trimesters, and malaria during pregnancy.

### Statistical analysis

The data analysis in the current study was performed in four steps using Stata/SE 13.0 for Windows (StataCorp LP, College Station, TX 77,845, USA). In the first step, descriptive statistics were performed. Then, cross-tabulations were used to examine the differences between women exposed to prenatal IPV and those not exposed, based on selected sociodemographic and economic characteristics of the respondents. Univariate and multivariable logistic regressions were performed to explore associations between prenatal exposure to IPV and LBW, the outcome of interest. The odds ratio (OR) and adjusted odds ratio (AOR), along with their respective 95% confidence intervals (CIs), are reported. The Pearson Chi-square goodness-of-fit test was utilised to evaluate the fit of the adjusted model.

### Ethical considerations

The Ghana Health Service’s Ethical Review Committee in Accra, Ghana, approved the study (GHS-ERC 033/11/22). In addition, permission was obtained from the Tamale Metropolitan Health Directorate and the heads of the health care facilities that participated in the study. Before collecting data, all respondents were informed of the objectives, risks, and potential benefits of the study and provided their written consent. To guarantee privacy, identifying information was deleted prior to data analysis. Minors or women younger than the consenting age of 18 years did not participate in the current study. All methods were performed in accordance with the Declaration of Helsinki.

## Results

Prevalence and distribution of self-reported intimate partner violence during pregnancy among the study sample.

**Fig. 2:**
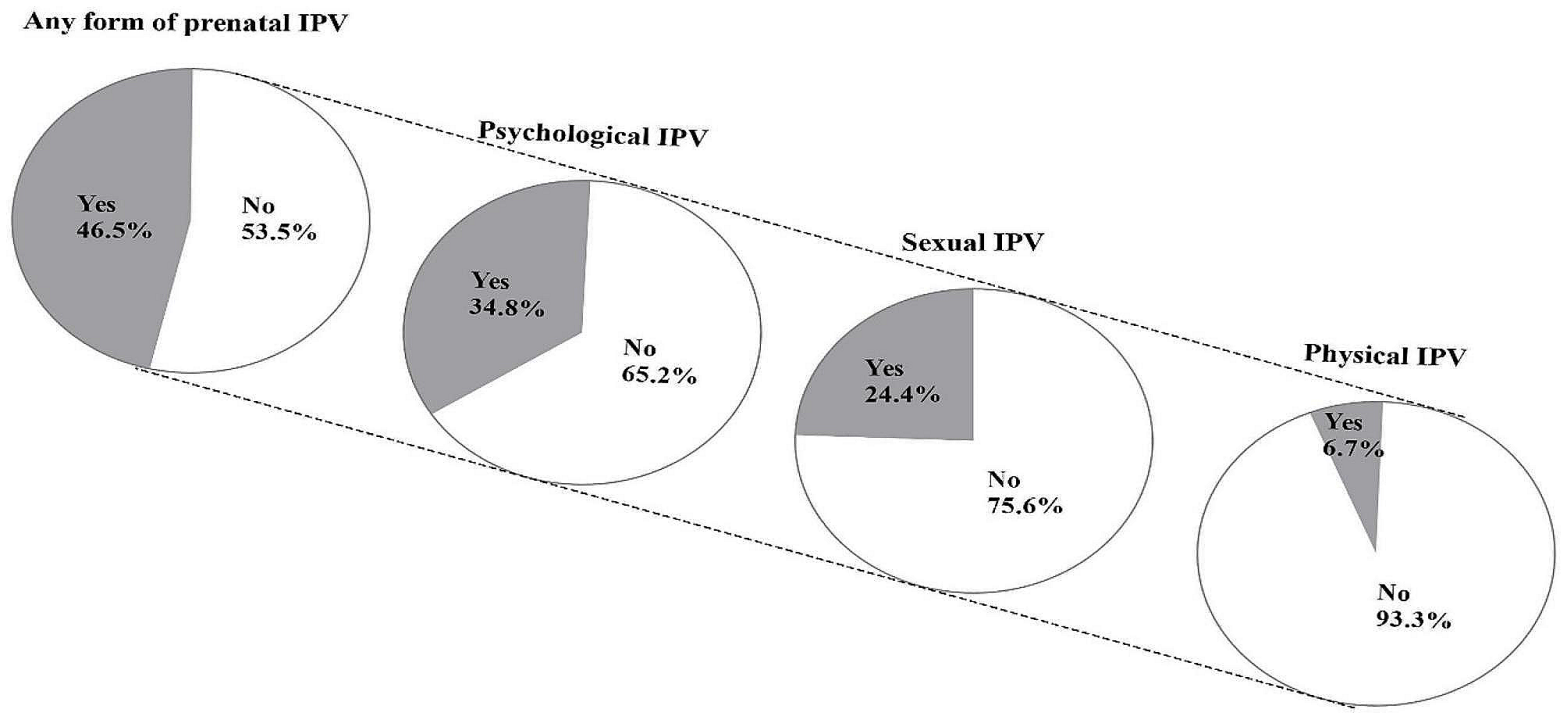
Prevalence of the various forms of intimate partner violence (IPV) during pregnancy (N = 402)

Figure 2 summarises the prevalence of each type of violence during pregnancy. It indicates that 46.5% (95% CI: 41.7, 51.4) of women in this sample experienced at least one type of IPV during their most recent pregnancy. Of these, 34.8% (95% CI: 30.3, 39.6) experienced psychological IPV, 24.4% (95% CI: 20.4, 28.8) experienced sexual IPV, and 6.7% (95% CI: 4.6, 9.6) experienced physical IPV. We went on to explore the percentage distribution of prenatal exposure to IPV based on selected sociodemographic and economic factors of the respondents. No significant differences were observed between women who reported being exposed to IPV during pregnancy and those who did not (Table 1).

**Table 1:** Percentage distribution of prenatal exposure to intimate partner violence by selected background characteristics of the study respondents (N = 402)

Variable	Frequency	Prenatal exposure to IPV		χ^2^ (*p*-value)
		No	Yes	
Age group				0.4256 (0.808)
15–24	47	24(51.1)	23(48.9)	
25–34	235	124(52.8)	111(47.2)	
35–49	120	67(55.8)	53(44.2)	
Received formal education				1.5735 (0.210)
No	258	144(55.8)	114(44.2)	
Yes	144	71(49.3)	73(50.7)	
Religion				0.2035 (0.652)
Christian	17	10(58.8)	7(41.2)	
Islam	385	205(53.2)	180(46.8)	
Ethnicity				1.6257 (0.654)
Dagomba	343	180(54.5)	163(47.5)	
Gonja	34	20(58.8)	14(41.2)	
Mamprusi	10	5(50.0)	5(50.0)	
Others	15	10(66.7)	5(33.3)	
Employment status				0.0065 (0.936)
Unemployed	96	51(53.1)	45(46.9)	
Employed	306	164(53.6)	142(46.4)	

IPV: intimate partner violence; Others: Frafra, Yoruba, Kusasi, Fulani, Dagaba, Akan

### Prevalence of low birth weight

In total, there were 36 newborns whose birth weights were indicative of LBW according to the standard classification used. This resulted in a prevalence of 9.0% (95% CI: 6.5, 12.2) in the sample.

Crude and adjusted logistic regression of the association between prenatal exposure to intimate partner violence and low birth weight.

Table 2 provides the results of crude and adjusted analyses of the association between prenatal exposure to IPV and delivering LBW babies. Prenatal exposure to IPV was significantly associated with an elevated risk of LBW in the crude analysis. From the results, women who reported being exposed to IPV during their most recent pregnancy were twice as likely to deliver LBW babies compared to unexposed women (OR = 2.18; 95% CI: 1.07, 4.44, *p* < 0.05). The association between prenatal exposure to IPV and LBW remained significant after adjusting for potential confounding by demographic, socioeconomic, and health-related factors. The adjusted regression analysis showed that exposed women were twice as likely as unexposed women to deliver LBW babies (AOR = 2.42; 95% CI: 1.12, 5.26, *p* < 0.05). Birthweight was also found to be related to the number of ANC visits, anaemia in the first trimester, and the sex of the newborn. In particular, women with anaemia in the first trimester had a higher risk of delivering LBW babies (AOR = 3.47; 95% CI: 1.47, 8.23, *p* < 0.01). On the other hand, the odds of delivering LBW babies were reduced among women who had at least four ANC visits before delivery compared to those who had fewer than four visits (AOR = 0.35; 95% CI: 0.14, 0.89, *p* < 0.05) and were lower among male newborns compared to their female counterparts (AOR = 0.23; 95% CI: 0.11, 0.49, *p* < 0.001).

**Table 2:** Crude and adjusted logistic regression of the association between prenatal exposure to intimate partner violence and low birth weight

Variable	Low birth weight	
	OR [95% CI]	AOR [95% CI]
Exposure to prenatal IPV		
No	1.00	1.00
Yes	2.18^*^[1.07,4.44]	2.42^*^[1.12,5.26]
Mother’s age group		
15–24		1.00
25–34		0.35[0.12,1.02]
35–49		0.76[0.17,3.41]
Received formal education		
No		1.00
Yes		0.84[0.37,1.94]
Employment status		
Not employed		1.00
Employed		0.95[0.40,2.25]
Religious affiliation		
Christian		1.00
Islam		1.15[0.13,9.97]
Gravida		0.70[0.47,1.05]
Timing of first antenatal care visit		
First trimester		1.00
Second trimester and beyond		0.51[0.19,1.40]
Number of antenatal care visits before birth		
Less than four		1.00
Four or more		0.35^*^[0.14,0.89]
Malaria during pregnancy		
No		1.00
Yes		0.97[0.40,2.35]
Anaemia in the first trimester		
No		1.00
Yes		3.47^**^[1.47,8.23]
Anaemia in the second trimester		
No		1.00
Yes		1.34[0.58,3.13]
Sex of newborn		
Female		1.00
Male		0.23^***^[0.11,0.49]
Adjusted model fitness test		
Number of observations		402
Number of covariate patterns		332
Pearson chi2 (318)		318.71
Prob > chi2		0.478

AOR: adjusted odds ratio; IPV: intimate partner violence; OR: odds ratio

^*^p < 0.05, ^**^p < 0.01, ^***^p < 0.001

## Discussion

In our study, the prevalence of IPV during pregnancy was high (47%), but within the ranges reported in the existing literature. Specifically, according to a study in Ethiopia, approximately 45% of women self-reported experiencing IPV during their most recent pregnancy [50]. In Tanzania, similarly, around three out of ten (30.3%) pregnant women experienced some kind of IPV from their intimate partner [17]. A study of postpartum mothers in Leon, Mexico, found that approximately 44% of women suffered violence during their pregnancy [20]. However, a cohort study in Spain found that 21.3% of pregnant women reported experiencing IPV [10]. Differences in study methodologies, context, and measures of IPV may explain the differences in prevalence rates among studies. In particular, we observed that studies that used multiple items to measure violence and report on a variety of various types of violence had the greatest prevalence rates [17, 20, 50, 51]. Our data showed that psychological or emotional IPV was more common in pregnant African women than other types of IPV, which supports previous research [12, 17, 51].

The results of the present study support our hypothesis that IPV exposure during pregnancy increases the risk of LBW in women. We found that, comparatively, women who were abused during their most recent pregnancy had a twofold greater risk of delivering LBW babies. Other researchers found that pregnant women who are victims of intimate partner abuse have a higher risk of delivering LBW babies compared with non-victims, which is consistent with the findings of our study [16, 52, 53]. However, our findings contradict earlier reports, which found no association between prenatal IPV exposure and birth outcomes [19–21]. A web of pathways could explain the association between prenatal IPV and LBW in the current study. Abuse during pregnancy may result in LBW due to pregnancy-related complications from direct assault and sexually transmitted infections, both of which are common among abused women [10, 11, 35]. The physiological response to stress experienced by abused pregnant women may aggravate pre-existing medical conditions, change eating habits, and influence prenatal health-seeking behaviour, thereby raising the risk of LBW among abused pregnant women [13, 37, 38, 43].

Nonetheless, the findings have some policy implications. WHO recommendations on ANC for a positive pregnancy advise that when assessing conditions that may be caused or made worse by IPV, clinical questions about the possibility of IPV should be asked during ANC visits [54]. A significant proportion of women in the study setting suffer IPV during pregnancy, which can be debilitating for both the mother and the unborn baby. In fact, more than two out of every five pregnant women in the Tamale Metropolitan Area of the northern region of Ghana are at risk of obstetric complications, premature rupture of membranes, inadequate maternal weight gain, underutilization of prenatal care services, and poor overall psychosocial health during pregnancy and postpartum, due to the direct and indirect effects of exposure to IPV [10, 11, 15, 40, 55]. In addition to being born with LBW, children born from these women are more likely to be born prematurely, to die within the first month of life, or to suffer nutritional and mental complications in childhood [56–58]. Indeed, neonatal complications are more common among women who experienced psychological violence during pregnancy [20], which was found to be more prevalent in the study population than the other types of violence. Our findings highlight the importance of detecting IPV in pregnant women in order to provide appropriate interventions. To be more specific, every pregnant woman should be screened for IPV at least once during each trimester, and those who have experienced violence should be closely monitored for weight gain and foetal growth to ensure a successful pregnancy and a healthy newborn.

Other findings of the present study need to be highlighted. We found that women who had at least four ANC visits prior to delivery and male neonates had a lower risk of LBW. Women who make fewer visits are more likely to miss out on the effective interventions provided during ANC for preventing and managing health-related conditions that may potentially impede the proper development of the unborn baby, such as anaemia, hypertensive disorders of pregnancy, and STIs, all of which increase the risk of LBW among women [59]. Some authors argue the gender difference in birthweight is due to gender-specific genes impacting insulin sensitivity, with the female foetus being less responsive to the trophic effects of insulin and therefore smaller [60].

Women who were anaemic in the first trimester had a greater risk of LBW. According to a systematic review and meta-analysis, approximately 42% of women in LMICs experience anaemia during pregnancy, and 12% of LBW is attributed to maternal anaemia [61]. Maternal iron depletion increases the risk of iron deficiency in the first three months of pregnancy, and maternal complications, including gestational hypertension and preeclampsia, are more likely among women with anaemia, resulting in an increased risk of LBW [62].

### Strengths and limitations

A key strength of the present study is that a representative sample was randomly selected from the eligible population, enabling the ability to generalise the findings to the study population. However, the use of a cross-sectional design in the current study limits our ability to draw causal conclusions. However, since the exposure occurred before the outcome, we can postulate that IPV during pregnancy is a substantial risk factor for adverse newborn outcomes, specifically LBW in the study setting. Moreover, the cross-sectional design was cost-effective, quick to conduct, and appropriate for achieving the objectives of the present study by providing a snapshot of the problem in the study area [63]. The study may also suffer from response bias because IPV is a socially stigmatised behaviour, which may limit the disclosure of IPV [64]. The factors included in the present study as covariates are not exhaustive. Other factors, such as body mass index, history of abortion, human immunodeficiency virus infection, and pollution, can also influence adverse birth outcomes [65–68]. Nonetheless, they were not included in the present study. As a result, the findings of the present study should be interpreted taking into account this limitation.

## Conclusions

IPV during pregnancy is common in the study setting, with psychological IPV being more widespread than other forms. LBW was more common among pregnant women who experienced IPV than among those who did not. It is vital to include questions about domestic violence in antenatal care protocols in health care facilities. Specifically, every pregnant woman should be screened for IPV at least once during each trimester, and those who have experienced violence should be closely monitored for weight gain and foetal growth in the study setting to avert the LBW associated with IPV.

### Electronic supplementary material

Below is the link to the electronic supplementary material.

Supplementary Material 1

## Data Availability

The dataset supporting this study’s conclusions is found in the publication. Nonetheless, the dataset is accessible upon reasonable request to the corresponding author.

## Abbreviations

ANC: Antenatal care
AOR: Adjusted odds ratio
CHPS: Community-based Health Planning and Services
CI: Confidence interval
DHS: Demographic and Health Survey
IPV: Intimate Partner Violence
IRB: Institutional Review Board
IUGR: Intrauterine Growth Restriction
LBW: Low birth weight
LMICs: Low and Middle-Income Countries
SSA: Sub-Saharan Africa
STIs: Sexually Transmitted Infections
WHO: World Health Organization
