# Supplementary material for: Magnitude of self-reported intimate partner violence against pregnant women in Ghana’s northern region and its association with low birth weight
Source: BMC Pregnancy Childbirth. 2024 Jan 4;24:29. doi: 10.1186/s12884-023-06229-6 (PMC10765694; doi:10.1186/s12884-023-06229-6)
Supplement: Supplementary file 1 — Supplementary Material 1 [file 12884_2023_6229_MOESM1_ESM.docx]

Additional file

Additional file 1: Responses to questions on physical, psychological, and sexual intimate partner violence

Table 1: Percentage distribution of responses to questions on physical and psychological intimate partner violence

| **Sno** | **Violence category** | **Frequency** | **Percentage** |
| --- | --- | --- | --- |
|  | **Physical violence** |  |  |
| 1 | Push you, shake, or throw something at you |  |  |
|  | No | 399 | 99.3 |
|  | Yes | 3 | 0.7 |
| 2 | Punch you with his fist or with something that could hurt you? |  |  |
|  | No | 393 | 97.8 |
|  | Yes | 9 | 2.2 |
| 3 | Kick you, drag you, or beat you up? |  |  |
|  | No | 398 | 99.0 |
|  | Yes | 4 | 1.0 |
| 4 | Try to choke you or burn you on purpose? |  |  |
|  | No | 402 | 100.0 |
|  | Yes | 0 | 0.0 |
| 5 | Threaten or attack you with a knife, gun or other weapon? |  |  |
|  | No | 402 | 100.0 |
|  | Yes | 0 | 0.0 |
| 6 | Twist your arm or pull your hair? |  |  |
|  | No | 382 | 95.0 |
|  | Yes | 20 | 5.0 |
|  | Number of acts of physical violence experienced |  |  |
|  | 0 | 375 | 93.3 |
|  | 1 | 20 | 5.0 |
|  | 2 | 5 | 1.2 |
|  | 3 | 2 | 0.5 |
|  | 4 | 0 | 0.0 |
|  | 5 | 0 | 0.0 |
|  | 6 | 0 | 0.0 |
|  | Exposed to any form of physical violence |  |  |
|  | No | 375 | 93.3 |
|  | Yes | 27 | 6.7 |
|  | **Psychological violence** |  |  |
| 1 | Say or do something to humiliate you in front of others? |  |  |
|  | No | 388 | 96.6 |
|  | Yes | 14 | 3.4 |
| 2 | Threatened to hurt or harm you or someone you care about? |  |  |
|  | No | 399 | 99.3 |
|  | Yes | 3 | 0.7 |
| 3 | Insult or made you feel bad about yourself? |  |  |
|  | No | 264 | 65.9 |
|  | Yes | 138 | 34.1 |
|  | Number of acts of psychological violence experienced |  |  |
|  | 0 | 262 | 65.2 |
|  | 1 | 127 | 31.6 |
|  | 2 | 11 | 2.7 |
|  | 3 | 2 | 0.5 |
|  | Exposed to any form of psychological violence |  |  |
|  | No | 262 | 65.2 |
|  | Yes | 140 | 34.8 |
|  | **Sexual violence** |  |  |
| 1 | Physically force you to have sexual intercourse with him when you did not want to? |  |  |
|  | No | 305 | 75.9 |
|  | Yes | 97 | 24.1 |
| 2 | Physically force you to perform any other sexual acts you did not want to? |  |  |
|  | No | 379 | 94.3 |
|  | Yes | 23 | 5.7 |
| 3 | Force you with threats or in any other way to perform sexual acts you did not want to? |  |  |
|  | No | 305 | 75.9 |
|  | Yes | 97 | 24.1 |
|  | Number of acts of sexual violence experienced |  |  |
|  | 0 | 304 | 75.6 |
|  | 1 | 1 | 0.2 |
|  | 2 | 75 | 18.7 |
|  | 3 | 22 | 5.5 |
|  | Exposure to any form of sexual violence |  |  |
|  | No | 304 | 75.6 |
|  | Yes | 98 | 24.4 |
